# The Timing of Stimulation and IL-2 Signaling Regulate Secondary CD8 T Cell Responses

**DOI:** 10.1371/journal.ppat.1005199

**Published:** 2015-10-02

**Authors:** Shaniya H. Khan, Matthew D. Martin, Gabriel R. Starbeck-Miller, Hai-Hui Xue, John T. Harty, Vladimir P. Badovinac

**Affiliations:** 1 Interdisciplinary Graduate Program in Immunology, University of Iowa, Iowa City, Iowa, United States of America; 2 Department of Microbiology, University of Iowa, Iowa City, Iowa, United States of America; 3 Department of Pathology, University of Iowa, Iowa City, Iowa, United States of America; La Jolla Institute for Allergy and Immunology, UNITED STATES

## Abstract

Memory CD8 T cells provide protection to immune hosts by eliminating pathogen-infected cells during re-infection. While parameters influencing the generation of primary (1°) CD8 T cells are well established, the factors controlling the development of secondary (2°) CD8 T cell responses remain largely unknown. Here, we address the mechanisms involved in the generation and development of 2° memory (M) CD8 T cells. We observed that the time at which 1° M CD8 T cells enter into immune response impacts their fate and differentiation into 2° M CD8 T cells. Late-entry of 1° M CD8 T cells into an immune response (relative to the onset of infection) not only facilitated the expression of transcription factors associated with memory formation in 2° effector CD8 T cells, but also influenced the ability of 2° M CD8 T cells to localize within the lymph nodes, produce IL-2, and undergo Ag-driven proliferation. The timing of stimulation of 1° M CD8 T cells also impacted the duration of expression of the high-affinity IL-2 receptor (CD25) on 2° effector CD8 T cells and their sensitivity to IL-2 signaling. Importantly, by blocking or enhancing IL-2 signaling in developing 2° CD8 T cells, we provide direct evidence for the role of IL-2 in controlling the differentiation of Ag-driven 2° CD8 T cell responses. Thus, our data suggest that the process of 1° M to 2° M CD8 T cell differentiation is not fixed and can be manipulated, a notion with relevance for the design of future prime-boost vaccination approaches.

## Author Summary

Since memory CD8 T cells afford hosts increased protection, extensive research has been devoted to understanding the parameters that affect the generation of these cells. Humans are typically infected with pathogens more than once, thus leading to re-stimulation of existing primary memory CD8 T cell populations. The factors influencing the development of CD8 T cells responding to repetitive antigen stimulations remain unknown. We demonstrate that the time at which primary memory CD8 T cells encounter antigen and are re-stimulated during infection influences the outcome of a secondary pathogen-specific CD8 T cell response. We show that at the time of antigen re-encounter, interleukin-2 cytokine signals received by developing secondary CD8 T cells impact the rate of acquiring secondary memory CD8 T cell characteristics. These data indicate that secondary memory CD8 T cell generation is a process that can be manipulated, which may have implications in the development of consecutive prime-boost immunization strategies.

## Introduction

Memory CD8 T cells are an important component of the adaptive immune response because of their ability to establish long-lasting protective immunity against recurrent infections [1–6]. Memory CD8 T cells are derived from naïve Ag-specific CD8 T cells that responded to pathogen-derived Ags, underwent robust proliferative expansion, and survived the contraction phase [7,8]. The protection afforded by memory CD8 T cells is due to persistence at higher numbers, unique trafficking abilities and localization in peripheral tissues, and rapid initiation of effector functions after Ag re-encounter [1,9,10]. These characteristics of primary memory (1° M) CD8 T cells distinguish them from the naïve CD8 T cells they are derived from.

Research devoted to understanding the development of memory CD8 T cells suggests that the generation of 1° M CD8 T cells is influenced by a number of factors [2,11–13]. For instance, studies have shown that the number of 1° M CD8 T cells generated correlates with the number of accumulated 1° effector CD8 T cells at the peak of expansion [14,15]. Therefore, parameters influencing 1° effector CD8 T cell expansion and/or survival (e.g. Ag presentation, co-stimulation, and signal 3 cytokines) impact the generation of 1° M CD8 T cells [2,10–12]. Interestingly, these factors have also been shown to influence the rate of 1° M CD8 T cell differentiation [10,11,16]. As an example, naïve CD8 T cells activated in a low-inflammatory environment (e.g. peptide-coated DC vaccination) undergo reduced levels of proliferative expansion but acquire long-term memory characteristics at an accelerated rate [17–19]. Additionally, the modulation of functional Ag presentation (e.g. antibiotic treatment to stop bacterial infection) also impacts the transition of Ag-specific CD8 T cells from effector to memory cells [16,20,21]. Furthermore, naive CD8 T cells activated in the presence of pre-existing memory CD8 T cells of an unrelated Ag specificity acquire memory characteristics at an accelerated rate [22]. Finally, recruitment of naïve Ag-specific CD8 T cells over time into an immune response influences memory CD8 T cell differentiation based on when cognate Ag is encountered [23,24]. This suggests that the process of naïve to 1° M CD8 T cell differentiation is not fixed and that the progression to memory can be manipulated.

Studies have shown that the generation of large numbers of memory CD8 T cells enhances CD8 T cell-mediated protection to re-infection. An effective strategy to increase the absolute numbers of memory CD8 T cells is through prime-boost vaccinations that elicit 2° immune responses [25,26]. Recent studies from our lab have shown that repeated stimulations of Ag-specific CD8 T cells results in differential regulation of a large number of genes in subsequent populations of memory CD8 T cells [27]. Interestingly, similar to 1° M CD8 T cell responses, 2° M CD8 T cell numbers and phenotype are modulated by the type and duration of infection and levels of inflammation [28,29]. However, it has also been previously documented that 2° M CD8 T cells are slower to acquire a long-term memory phenotype compared to 1° M CD8 T cells [30].

Since the timing of stimulation of naïve CD8 T cells has been shown to influence 1° M CD8 T cell differentiation, we devised a model in which 1° M CD8 T cells are recruited at different times into the response relative to the initiation of infection to determine if the timing of recruitment influences the development of 2° CD8 T cell responses. IL-2 signaling impacts the accumulation and differentiation of 1° effector CD8 T cells [31,32], thus we addressed whether the timing of stimulation of 1° M CD8 T cells modulates sensitivity to IL-2 signaling, thereby affecting 2° expansion and 2° CD8 T cell differentiation. By regulating IL-2 signaling in developing 2° CD8 T cells, either by enhancing signaling with stimulatory IL-2 complexes or blocking IL-2 with a neutralizing antibody, we provided direct evidence of the contribution of this signaling mechanism in controlling the generation of 2° CD8 T cell responses. Overall, these data suggest that the process of 1° M to 2° M CD8 T cell generation and differentiation can be manipulated, which may have implications in the development of consecutive prime-boost immunization strategies.

## Results

### 1° M CD8 T cells are not recruited simultaneously after infection

The differentiation of 1° M CD8 T cells has been extensively studied, however much less is known about the factors that influence 2° M CD8 T cell generation. Previous studies have shown that the time at which naïve CD8 T cells recognize Ag during an immune response impacts the rate of acquiring 1° M CD8 T cell characteristics [23,24]. Therefore, we wanted to determine if the timing of stimulation of 1° M CD8 T cells was a factor impacting the generation of 2° M CD8 T cell responses.

**Fig 1:**
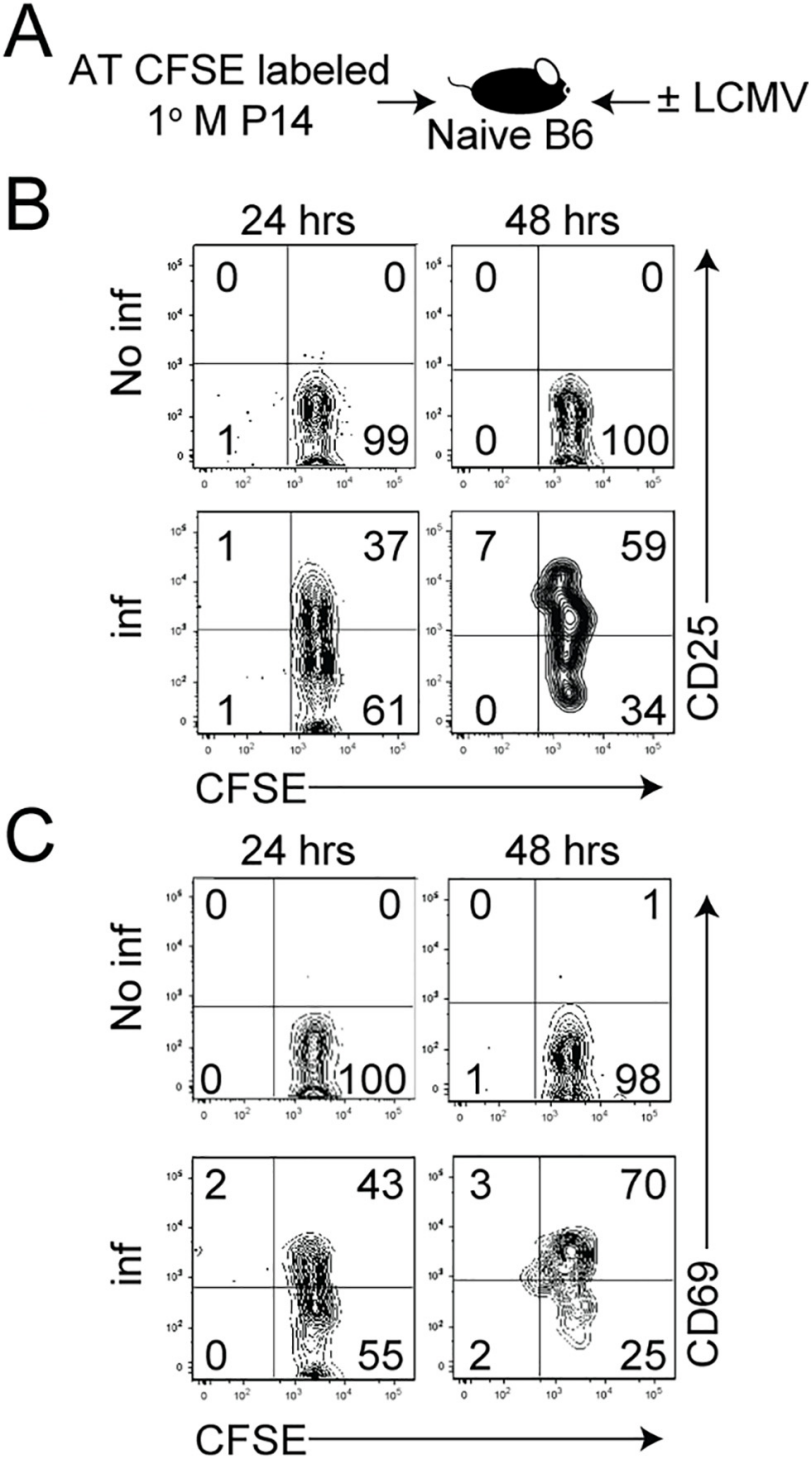
1° M CD8 T cells are not recruited simultaneously after infection. **A)** Experimental design. CFSE-labeled 1° M Thy1.1 P14 CD8 T cells (2x10^6^ cells/mouse, i.v.) were adoptively transferred into naïve B6 Thy1.2 recipient mice before LCMV infection. A non-infected group of recipient mice served as a control. The expression of **B)** CD25 and **C)** CD69 on CFSE-labeled 1° M P14 CD8 T cells isolated from the spleen of mice 24 and 48 hours after infection was determined. Numbers indicate the percentage of cells positive for the indicated molecule. Representative profiles of 3 mice per group are shown. Experiments in panels A-C are representative of 3 independent and similar experiments.

First, we asked if recruitment of 1° M CD8 T cells occurs simultaneously or over extended period of time after infection. To test this, LCMV-specific CFSE-labeled TCR-Tg 1° M P14 CD8 T cells (2x10^6^ cells/recipient; Thy1.1) [28,29] were transferred into naïve C57BL/6 (B6; Thy1.2/1.2) recipient mice that were either left uninfected or infected with the Armstrong strain of LCMV (Fig 1A, experimental design). The expression of CD25 and CD69, molecules known to be rapidly unregulated on activated CD8 T cells, was determined on responding 1° M P14 CD8 T cells. By 24 hours p.i., a fraction of 1° M CD8 T cells were found to be activated in the spleen, as determined by the upregulation of CD25 and CD69 (Fig 1B and 1C). By 48 hours p.i., the percentage of 1° M CD8 T cells that had responded to the infection and expressed these markers had increased; however, most of these responding cells were not undergoing division, as they still expressed high levels of CFSE. Interestingly, at this time there were still 1° M CD8 T cells that had not upregulated either activation marker, suggesting that similar to naïve Ag-specific CD8 T cells [23], 1° M CD8 T cells are not recruited simultaneously after infection.

To understand how the time at which 1° M CD8 T cell recognize Ag in an immune response might impact the differentiation of 2° CD8 T cell responses, we modified a model previously used to determine how time of the recruitment of naïve CD8 T cells influences their development into a 1° M CD8 T cell pool [23,24]. In preliminary experiments using our modified approach, we wanted to first confirm that naïve CD8 T cells recognizing Ag late in an immune response rapidly acquire 1° M characteristics. Physiologically low number of naïve P14 CD8 T cells (5x10^3^ cells/recipient; Thy1.1 [33]) were adoptively transferred into naïve B6 (Thy1.2/1.2) recipient mice on the same day (‘early’ group) or 3 days (‘late’ group) after LCMV infection (S1A Fig, experimental design). Staggering infections in this adoptive transfer model enabled us to track the differentiation of 1° Ag-specific CD8 T cell responses in both groups of mice at the same time relative to the day of transfer. In the ‘late’ group, Ag-specific CD8 T cells are prevented from early priming because they are transferred into mice 3 days after infection. As seen previously, naïve CD8 T cells that recognized Ag late in the immune response underwent reduced levels of proliferative expansion (S1B Fig) [23]. However, despite limited expansion and persistence at lower numbers over time (S1C and S1E Fig), these Ag-specific CD8 T cells stimulated late displayed a memory phenotype at an accelerated rate ((increase in CD27 and CD62L and decrease in KLRG1) S1D and S1F Fig). Consistent with the notion that late-stimulation in the immune response does not influence rates of contraction [23], we found that relative to the respective peak of expansion of activated CD8 T cells in ‘early’ and ‘late’ groups, similar proportions of 1° effector CD8 T cells survived the contraction phase (S1G Fig). Similar results were obtained using naive TCR-Tg OT-I CD8 T cells and *L*. *monocytogenes* infection (S1H Fig).

### The magnitude of proliferative expansion and transcriptional programming of 2° effector CD8 T cells is impacted by the timing of stimulation of 1° M CD8 T cells

**Fig 2:**
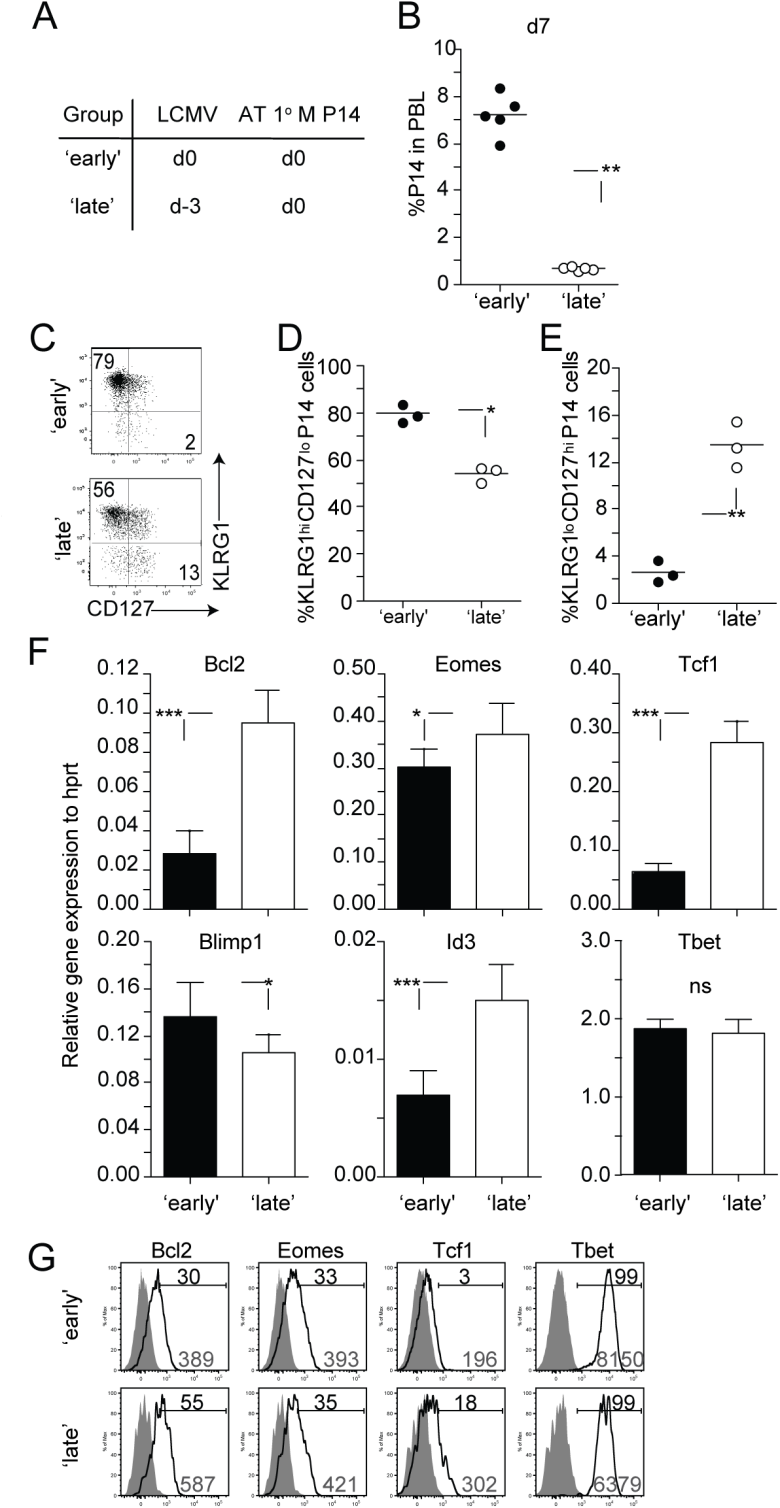
Timing of stimulation impacts proliferative expansion and transcriptional program of 2° effector CD8 T cells. **A)** Experimental design. Naïve B6 Thy1.2/1.2 mice received a transfer of 1° M Thy1.1 P14 CD8 T cells (2x10^4^ cells/mouse, i.v.) on the day of (‘early’ group) or 3 days after (‘late’ group) infection with LCMV (2x10^5^ PFU/mouse i.p.). **B)** The percentage of 2° effector P14 CD8 T cells in the PBL at day 7 after transfer. Dots represent individual mice and the line represents the mean. **C)** Representative dot plots showing the expression of KLRG1 and CD127 molecules on 2° effector P14 CD8 T cells isolated from the spleen at day 7 after transfer. The percentage of 2° effector P14 CD8 T cells expressing a **D)** KLRG1^hi^ CD127^lo^ or **E)** KLRG1^lo^ CD127^hi^ phenotype. **F)** Total RNA was extracted from 2° effector P14 CD8 T cells and analyzed for the expression of indicated transcripts using quantitative RT-PCR. Relative expression to Hprt is shown. The data are mean + SD of triplicate measurements of a total of three samples from each group. G) Representative histograms showing the expression of the molecules Bcl2, Eomes, Tcf1, and Tbet on 2° effector P14 CD8 T cells from spleens of mice from ‘early’ and ‘late’ groups. Shaded graphs represent isotype control staining and open graphs represent specific Ab staining on gated 2° effector Thy1.1 P14 CD8 T cells. Black numbers indicate the percentage of P14 CD8 T cells positive for indicated markers and grey numbers indicate gMFI of P14 CD8 T cells. Data are of 3–5 mice per group and experiments are representative of 2–3 independent experiments. The p values are indicated.

Next we explored the extent to which timing of stimulation influenced the development of 2° CD8 T cell responses. To test this, 1° M P14 CD8 T cells (2x10^4^ cells/recipient; Thy1.1) [28,29] were transferred into naïve B6 (Thy1.2/1.2) recipient mice on the same day (‘early’ group) or 3 days after (‘late’ group) LCMV infection (Fig 2A, experimental design). Examination of P14 CD8 T cells in the blood day 7 after transfer revealed that the magnitude of 2° expansion was significantly decreased in mice in the ‘late’ group (Fig 2B). This suggests that the time at which 1° M CD8 T cells encounter Ag in an immune response impacts the accumulation of 2° effector CD8 T cells.

After 1° infection with intracellular pathogens such as LCMV, subsets of differentiating 1° effector CD8 T cells can be distinguished based on the expression of phenotypic markers like KLRG1 and CD127. For example, CD8 T cells exhibiting a KLRG1^low^ CD127^hi^ phenotype at the peak of a 1° anti-LCMV immune response have increased potential to populate the memory CD8 T cell pool [34–36]. Additionally, studies have shown that transcription factors, that play a crucial role in the differentiation of 1° CD8 T cell responses [37,38], are differentially regulated in Ag-specific CD8 T cells based on signals received at early stages of activation. Interestingly, we found that a greater percentage of 2° effector CD8 T cells within the ‘late’ group expressed a KLRG1^low^ CD127^hi^ phenotype (Fig 2C–2E). The mRNA expression of transcription and pro-survival factors within these 2° effector CD8 T cells in mice within the ‘late’ group correlated with this phenotype. Expression of Eomes, Tcf1, and Id3, transcription factors associated with memory formation was significantly increased in 2° effector CD8 T cells derived from the ‘late’ group (Fig 2F) [35,39–42]. Conversely, the expression of Blimp1, known to promote terminal differentiation of CD8 T cells [43], was significantly decreased in these 2° effector CD8 T cells. Finally, expression of the pro-survival factor Bcl2 was increased in CD8 T cells recruited late into the response (Fig 2F). The protein expression of some (Bcl2, Tcf1, and Tbet) but not all (Eomes) transcription and pro-survival factors correlated with levels of mRNA on 2° effector CD8 T cells in ‘early’ and ‘late’ groups, (Fig 2G). Our data suggests that the timing of stimulation of 1° M CD8 T cells influences the phenotypic and transcriptional programs of the developing 2° effector CD8 T cell pool.

### 1° M CD8 T cells stimulated late in the immune response progress to a long-term 2° M phenotype at an accelerated rate

**Fig 3:**
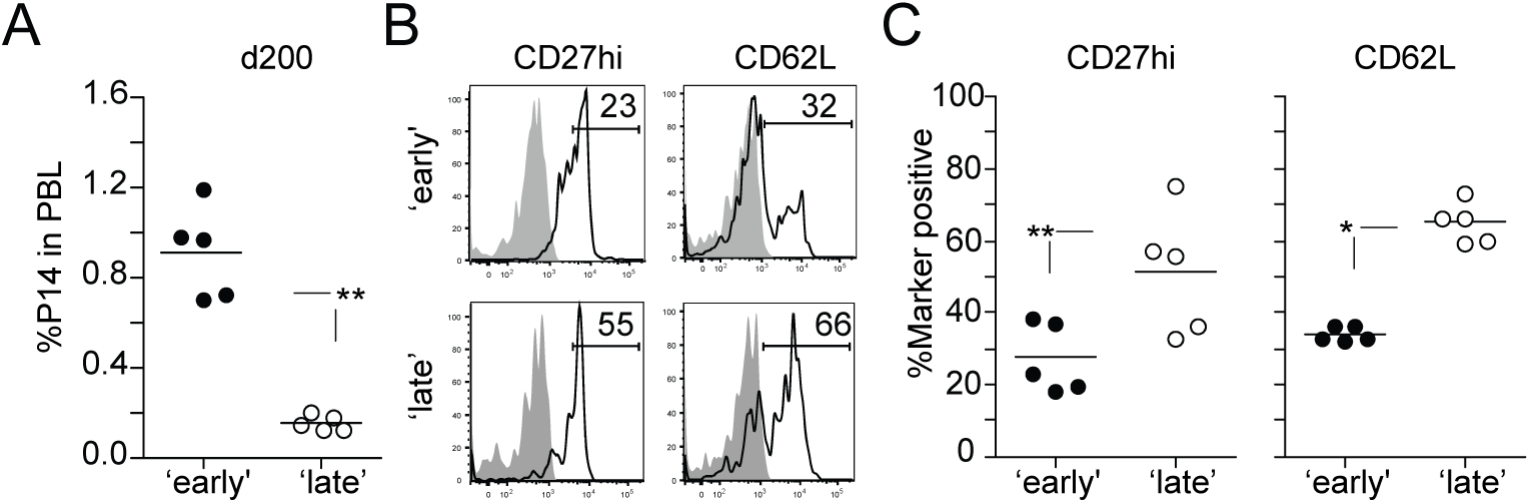
Late stimulated 1° M CD8 T cells acquire a long-term 2° M phenotype faster. **A)** The percentage of 2° M Thy1.1 P14 CD8 T cells in the PBL of individual mice at day 200 after transfer. **B)** Representative histograms showing the expression of the molecules CD27 and CD62L on 2° M P14 CD8 T cells in the PBL. Shaded graphs represents isotype control staining and open graphs represent specific Ab staining on gated 2° M Thy1.1 P14 CD8 T cells. **C)** The percentage of 2° M P14 CD8 T cells positive for CD27 and CD62L. Dots represent individual mice and the line represents the mean. Data are of 5 mice per group and this experiment was repeated twice with similar results. The p values are indicated.

Although the differentiation state of effector CD8 T cells at the peak of an immune response provides insight on the ability of these cells to populate the memory pool, the generation and maintenance of memory CD8 T cell responses is a time-dependent process. Furthermore, with each additional Ag encounter the rate at which populations of activated CD8 T cells acquire memory characteristics slows substantially [2,44–47]. To extend our observation that the timing of stimulation of 1° M CD8 T cells influences the phenotypic programming of 2° CD8 T cell responses, we determined the fate of 2° M CD8 T cells in ‘early’ and ‘late’ groups at a memory time-point (day 200 after transfer). The number of 2° M CD8 T cells in both groups of mice reflected the initial degree of 2° expansion, as 2° M CD8 T cells persisted at lower frequencies in the PBL of mice in the ‘late’ group (Fig 3A). However, late-entry of 1° M CD8 T cells in an immune response facilitated faster re-expression of the molecules CD27, CD62L and KLRG-1 during 2° M CD8 T cell development (Figs 3B and 3C and S2). To determine the generality of this data, we also employed a model of systemic bacterial infection. 1° M OT-I CD8 T cells (4x10^4^ cells/recipient; Thy1.1) were transferred into naïve B6 (Thy1.2) recipient mice on the same day (‘early’ group) or 3 days after (‘late’ group) infection with attenuated Ova-expressing *L*. *monocytogenes* (Att LM-Ova) (S3A Fig, experimental design). Similar to the findings observed with P14 TCR-Tg CD8 T cells and LCMV infection, we found that the timing of stimulation of 1° M OT-I CD8 T cells impacted 2° expansion, numbers of generated 2° M CD8 T cells, and rate of expression of 2° M phenotypic markers (S3B–S3D Fig).

Taken together, these data suggest that the time at which 1° M CD8 T cells recognize Ag during an immune response impacts the rate at which long-term 2° M characteristics are acquired.

### The timing of stimulation modulates the function of 2° M CD8 T cells

**Fig 4:**
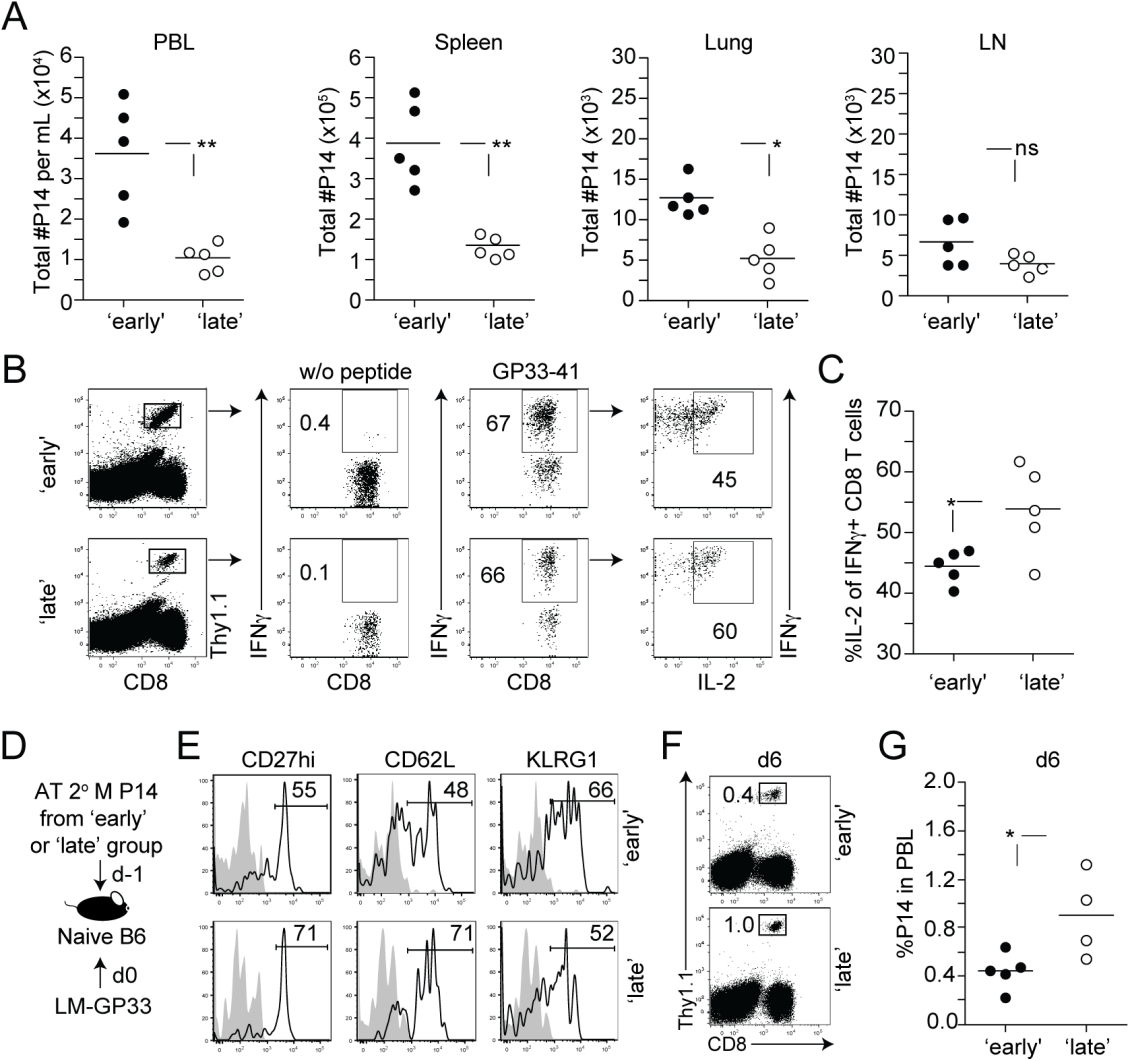
The timing of stimulation modulates the function of 2° M CD8 T cells. **A)** Total numbers of 2° M Thy1.1 P14 CD8 T cells in mL of PBL, and in the spleen, lung, and LN of individual mice from ‘early’ and ‘late’ group of mice 7 months after the initiation of the experiment. **B)** Representative dot plots showing cytokine production by 2° M P14 CD8 T cells, isolated from the spleen of individual mice after short *ex vivo* incubation in the presence of GP33 peptide. Numbers represent the percentage of 2° M P14 CD8 T cells that were positive for IFNγ and IL-2. **C)** Percentage of IFNγ producing 2° M P14 CD8 T cells that co-produce IL-2. Dots represent individual mice and the line represents the mean. **D)** Experimental design, 2° M Thy1.1 P14 CD8 T cells from ‘early’ and ‘late’ groups of mice were isolated on day 260 after transfer by positive selection and transferred in equal numbers (1.5x10^4^ cells/mouse, i.v.) into naïve B6 Thy1.2/1.2 recipients 1 day before Att LM-GP33 (1x10^7^ CFU/mouse i.v.) infection. **E)** Representative histograms showing the expression of the molecules CD27, CD62L, and KLRG1 on transferred 2° M P14 CD8 T cells from ‘early’ and late’ groups of mice. Shaded graphs represent isotype control staining and open graphs represent specific Ab staining on gated 2° M Thy1.1 P14 CD8 T cells. **F)** Representative dot plots showing 3° expansion of P14 CD8 T cells on day 6 after infection with Att LM-GP33. Numbers indicate the percentage of P14 CD8 T cells in the PBL. **G)** Percentage of P14 CD8 T cells in the PBL of individual mice on day 6 is shown. Dots represent individual mice and the line represents the mean. Data are of 5 mice per group and representative of 2–3 independent experiments. The p values are indicated; ns- not significant.

To address whether the timing of stimulation of 1° M CD8 T cells impacts the function of 2° M CD8 T cells, the tissue distribution of 2° M P14 CD8 T cells in 2° lymphoid organs and tertiary (3°) tissues of mice in ‘early’ and ‘late’ groups 200 days after transfer was first determined. There was a statistically significant decrease in the number of 2° M P14 CD8 T cells recovered from the PBL, spleen, and lung of mice in the ‘late’ group (Fig 4A). In contrast, increased levels of CD62L expression (Fig 3C) allowed 2° M CD8 T cells in the ‘late’ group to preferentially home to the LN (Fig 4A), suggesting that the timing of stimulation impacts the localization of 2° M CD8 T cells. In addition to changes in tissue distribution, the function of 2° M P14 CD8 T cells in ‘early’ and ‘late’ groups was evaluated after *ex vivo* peptide stimulation. A similar percentage of 2° M CD8 T cells from both groups of mice produced IFNγ (Fig 4B), however 2° M CD8 T cells in the ‘late’ group had a significantly increased ability to co-produce IL-2 (Fig 4C). Given that the timing of stimulation was found to modulate a rapid polyfunctional cytokine response after peptide stimulation, a defining characteristic of memory, we were also interested in determining whether Ag-driven 3° expansion was also modulated. To test this on a per-cell basis, equal numbers (1.5x10^4^ cells/recipient; Thy1.1) of 2° M P14 CD8 T cells from ‘early’ and ‘late’ groups were transferred into new naïve B6 Th1.2 hosts. One day after transfer, both groups of recipient mice were infected with an attenuated strain of *L*. *monocytogenes* expressing the LCMV-derived GP33 epitope (Att LM-GP33) (Fig 4D and 4E). Ag-driven 3° accumulation of P14 cells was significantly increased in the PBL of mice that received memory CD8 T cells from the ‘late’ group (Fig 4F and 4G), suggesting that the timing of stimulation also modulates their ability to undergo proliferative expansion in numbers upon additional Ag-encounter.

Taken together, these data show that 1° M CD8 T cells that recognized Ag late during a 2° immune response have an increased capacity to traffic to the LN, produce IL-2, and undergo robust proliferative expansion after Ag re-encounter.

### The timing of stimulation of 1° M CD8 T cells regulates IL-2 sensitivity and expression of cell-cycle related proteins

It is well established that early inflammatory signals received by activated CD8 T cells shape subsequent phases of the CD8 T cell response. For example, in response to signal 3 cytokines, either IL-12 or type I IFNs, activated CD8 T cells maintain high-affinity IL-2 signaling *in vivo* which results in continued expression of cell-cycle associated genes and extended cellular division [48]. In addition to driving optimal accumulation of activated CD8 T cells, IL-2 signaling has also been shown to regulate 1° M CD8 T cell differentiation. Based on the expression of the high-affinity IL-2 receptor α chain (CD25), activated CD8 T cells either favor terminal differentiation in response to strong IL-2 signaling or have an increased potential to become 1° M cells as a result of lower CD25 expression and diminished sensitivity to IL-2 signals [31,32].

**Fig 5:**
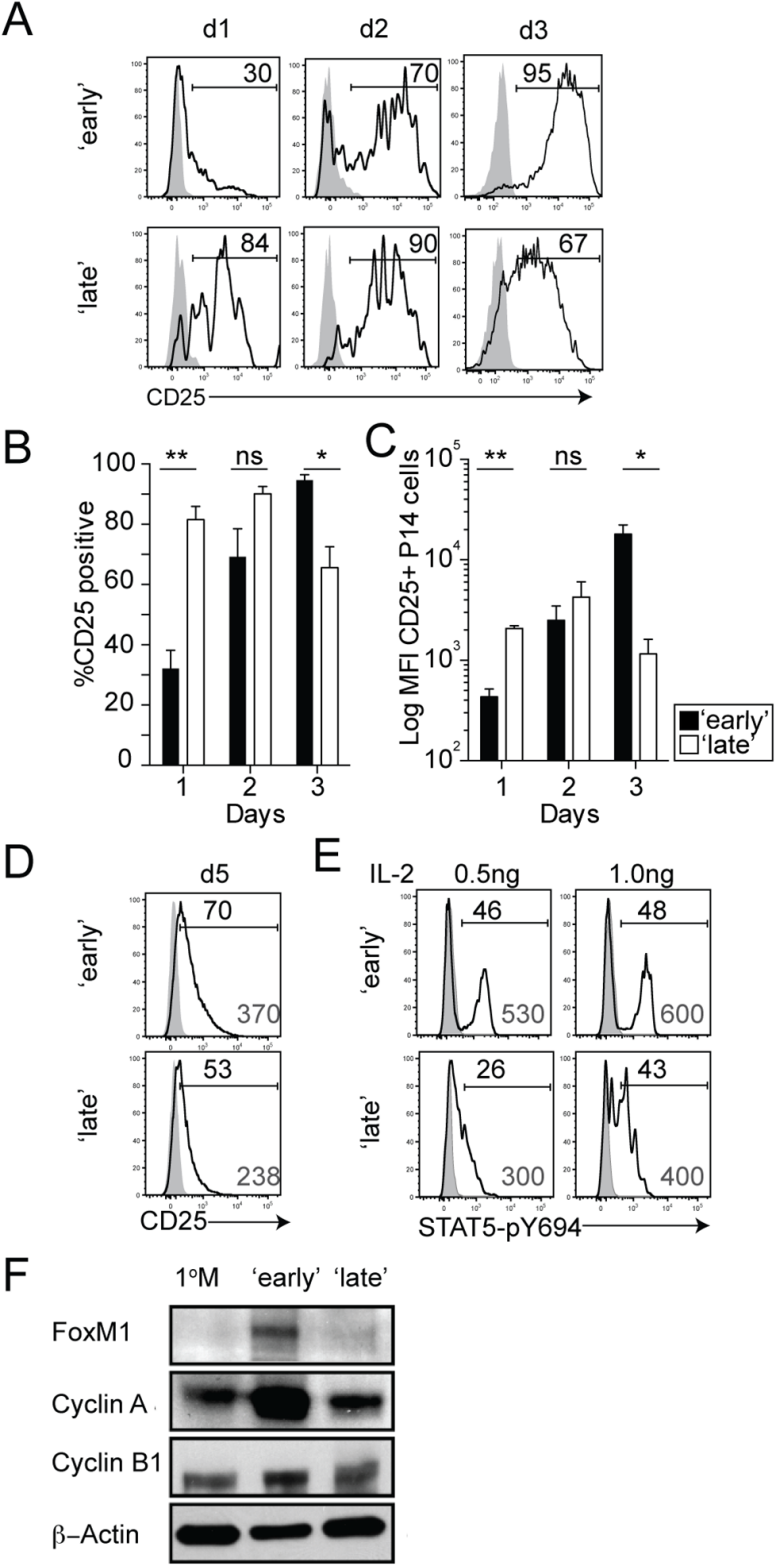
The timing of stimulation regulates IL-2 sensitivity and expression of cell-cycle related proteins. **A)** Representative histograms showing the expression of the CD25 molecule on 2° effector Thy1.1 P14 CD8 T cells isolated from the spleens of ‘early’ and ‘late’ groups of mice on indicated days after transfer. Shaded graphs represent isotype control staining and open graphs represent specific Ab staining on gated 2° effector Thy1.1 P14 CD8 T cells. Histograms are concatenated using FlowJo software from 1 of 2 independent experiments displaying equal representation from 3 individual mice. **B)** Percentage of 2° effector P14 CD8 T cells positive for CD25 on various days after transfer. Data are presented as mean+ SEM of 3 mice per group. **C)** Log geometric mean fluorescence intensity (gMFI) of CD25^+^ 2° effector P14 CD8 T cells. Data are presented as mean+ SEM of 3 mice per group. **D)** Histograms showing the expression of CD25 on 2° effector P14 CD8 T cells isolated from the spleen 5 days after transfer. Shaded graphs represent isotype control staining and open graphs represent staining on gated 2° effector Thy1.1 P14 CD8 T cells. Black numbers indicate the percentage of P14 CD8 T cells positive for CD25 and grey numbers indicate the gMFI of CD25^+^ P14 CD8 T cells. Histograms are concatenated using FlowJo software from one of two independent experiments displaying equal representation from 4–5 individual mice. **E)** STAT5-pY694 was measured in 2° effector P14 CD8 T cells in the absence (shaded histograms) or after 15 minutes of IL-2 stimulation (open histograms). **F)** Protein expression of Foxm1, Cyclin A, and Cyclin B1 in 2° effector (day 7) and 1° M P14 CD8 T cells. All experiments are representative of 2–3 independent experiments. The p values are indicated; ns-not significant.

We were interested in determining if 1° M CD8 T cells stimulated early or late in the immune response receive signals at the initial expansion phase that initiate unique programs of 2° M CD8 T cell differentiation. Therefore, we determined the extent to which the timing of stimulation of 1° M CD8 T cells affects the duration of expression of CD25 on 2° effector CD8 T cells (Fig 5A). Although an increased frequency of 2° effector P14 CD8 T cells expressed a CD25^hi^ phenotype in the ‘late’ group compared to the ‘early’ group on day 1 after transfer, this marked increase was transient (Fig 5B and 5C). Surface expression of CD25 gradually increased and was sustained on 2° effector P14 CD8 T cells in the ‘early’ group as late as 5 days after transfer (Fig 5A–5D), while expression decreased over time on 2° effector P14 CD8 T cells in the ‘late’ group. Furthermore, in response to stimulation with titrated amounts of recombinant IL-2 *in vitro*, isolated 2° effector P14 CD8 T cells from ‘early’ mice exhibited more pronounced STAT5 activation at lower concentrations of IL-2 compared to ‘late’ mice (Fig 5E). This suggests that the duration of CD25 expression on 2° effector CD8 T cells correlates with IL-2 sensitivity and is modulated by the timing of stimulation.

Activated 1° CD8 T cells that maintain high-affinity IL-2 signaling show continued expression of cell-cycle associated genes and proteins which drives enhanced proliferative expansion [48]. Since we found that 2° effector P14 CD8 T cells in ‘early’ groups of mice not only undergo increased levels of proliferative expansion (Figs 2B and S3B), but also sustain CD25 expression (Fig 5A–5D), we wanted to determine whether this increased sensitivity to IL-2 signaling enhances the expression of cell-cycle related proteins such as FoxM1, Cyclin A, and Cyclin B1. Expression of these proteins was higher in 2° effector P14 CD8 T cells in the ‘early’ group, which correlated with sustained IL-2 signaling and enhanced ability of these cells to undergo proliferative expansion (Fig 5F). Thus, these data suggest that the timing of stimulation of 1° M CD8 T cells impacts the strength of IL-2 signals received by differentiating 2° effector CD8 T cells as a consequence of differential CD25 expression.

### The availability of IL-2 signals impacts the differentiation of 2° CD8 T cells

**Fig 6:**
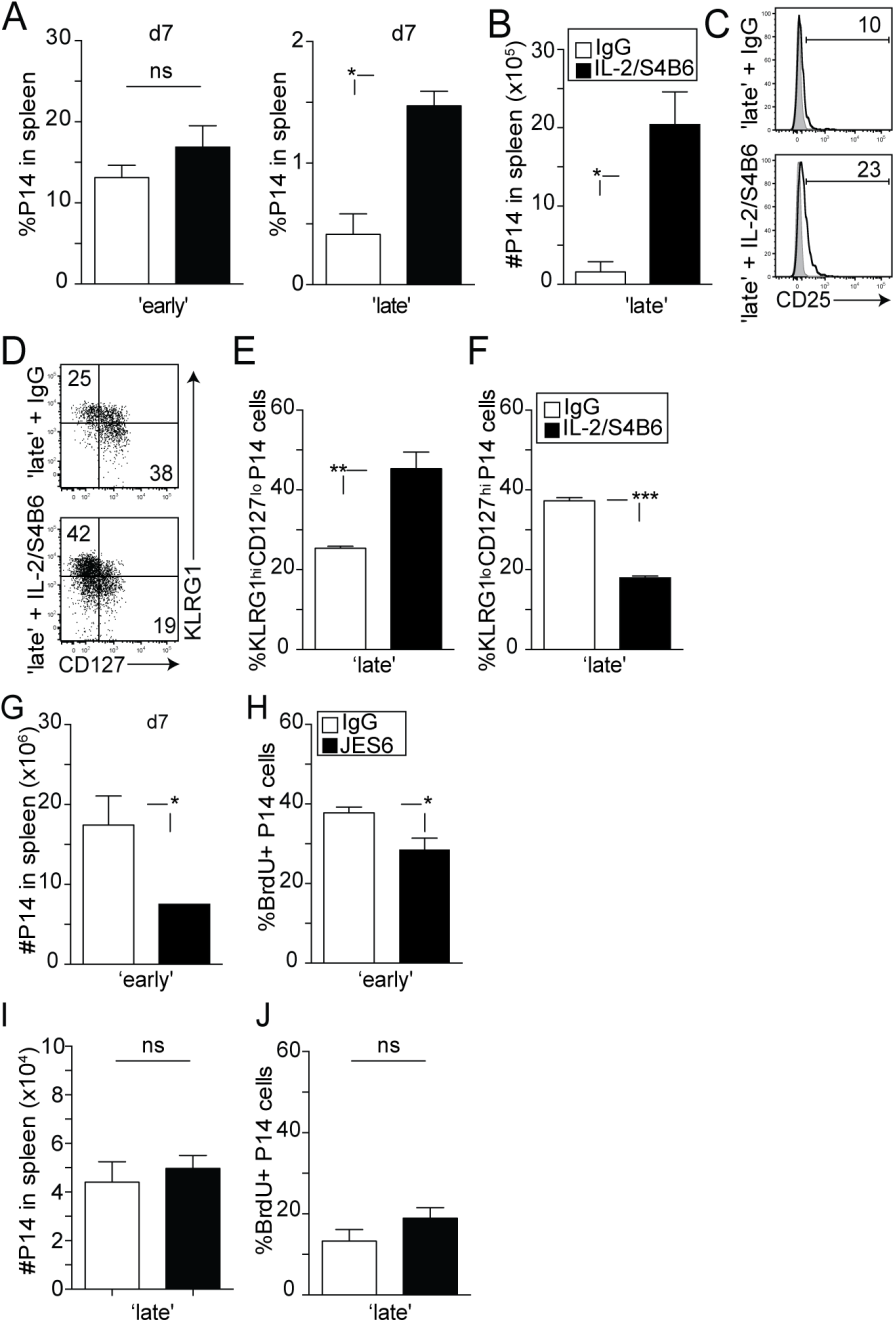
The availability of IL-2 signals impacts the differentiation of 2° CD8 T cells. ‘Early’ and ‘late’ groups of mice were treated with control IgG or high-affinity IL-2 receptor stimulating complex (1.5 μg/mL IL-2: 50μg/mL S4B6) from days 4–6 post-transfer. **A)** Percentage of 2° effector P14 CD8 T cells in the spleens of mice from ‘early’ and ‘late’ groups. **B)** Total numbers of 2° effector P14 Thy1.1 CD8 T cells from the spleens of individual mice in the ‘late’ group at day 7 post transfer. Data are presented as mean+ SEM of 3–4 mice per group. **C)** Representative histograms showing the expression of the CD25 molecule on 2° effector P14 CD8 T cells from the ‘late’ group. Shaded graph represents isotype control staining and open graphs represent specific Ab staining on gated 2° effector Thy1.1 P14 CD8 T cells. **D)** Representative dot plots showing the expression of KLRG1 and CD127 molecules on 2° effector P14 CD8 T cells at day 7 after transfer. The percentage of 2° effector P14 CD8 T cells expressing a **E)** KLRG1^hi^ CD127^lo^ or **F)** KLRG1^lo^ CD127^hi^ phenotype in the spleen of individual mice in the ‘late’ group is shown. Data are presented as mean+ SEM of 3–4 mice per group. **G-J)** ‘Early’ and ‘late’ groups of mice were treated with control IgG or IL-2 blockade (JES6-1A12, 500μg/mouse) from day 2–5 post-transfer. **G)** The total number of P14 CD8 T cells from the spleen of individual mice at day 7 after transfer. **H)** The percentage of BrdU^+^ P14 CD8 T cells in the spleen**. I)** The total number of P14 CD8 T cells from the spleens of individual mice at day 7 after transfer. **J)** The percentage of BrdU^+^ P14 CD8 T cells in the spleen. Data in **G-J)** are presented as mean + SEM of 4–5 mice per group. All experiments are representative of 2 independent experiments. The p values are indicated.

Since we found that the responsiveness of 2° effector CD8 T cells to IL-2 is dependent on the timing of stimulation, we wanted to determine whether providing sustained IL-2 signaling during early or late recruitment of 1° M CD8 T cells would impact the subsequent differentiation of 2° CD8 T cell responses. It has been previously demonstrated that the biological activity of the IL-2 cytokine is enhanced once bound to a particular anti-IL-2 monoclonal antibody [49]. Notably, the S4B6 clone binds to the region of IL-2 that interacts with the CD25 receptor chain [49]. Stimulatory IL-2/S4B6 antibody complexes therefore selectively target CD122hi cells, independently of CD25, and facilitate IL-2 signaling in memory CD8 T cells and NK cells [49]. During a secondary infection with LCMV, 2° effector P14 CD8 T cells in ‘early’ and ‘late’ groups of mice similarly expressed CD122 over time after transfer (S4 Fig). Therefore, we chose to use these complexes as a tool to provide sustained IL-2 signaling in developing secondary effector CD8 T cells. ‘Early’ and ‘late’ groups of mice were treated with IL-2/S4B6 antibody complexes at days 4–6 post transfer and the magnitude of the secondary expansion was determined in the spleen at day 7 post transfer. Interestingly, enhanced accumulation in the frequency (Fig 6A) and total numbers (Fig 6B) of 2° effector P14 CD8 T cells was only observed in the ‘late’ group of mice. This increase in accumulation also correlated with a modest increase in CD25 expression on 2° effector P14 CD8 T cells in the ‘late’ group (Fig 6C). These data suggest that since 2° effector CD8 T cells in the ‘early’ group are already sensitive to IL-2 signaling, IL-2 stimulation resulted in no additional effect; however, 2° effector CD8 T cells in the ‘late’ group underwent increased accumulation in numbers due to sustained IL-2 signaling provided by the IL-2/S4B6 antibody complexes.

It is important to note that overall increase in 2° CD8 T cell accumulation in the ‘late’ group is facilitated by both antigenic stimulation and increased sensitivity to IL-2 signals following complex treatment, as 1° M CD8 T cells activated with the stimulatory IL-2/S4B6 complexes in the absence of infection undergo a moderate level of homeostatic driven proliferation (S5 Fig). Interestingly, manipulating IL-2 signaling also resulted in an altered differentiation status in 2° effector P14 CD8 T cells of ‘late’ mice. Similar to what has been previously documented by Xue et al. [50], increased IL-2 signaling resulted in downregulated IL-7Rα expression (Fig 6D) in a large fraction of cells in the ‘late’ group, with 40–50% of 2° effector CD8 T cells now displaying a KLRG1^hi^CD127^lo^ phenotype after complex treatment (Fig 6E and 6F). These data suggest that increased sensitivity to IL-2 signals favors terminal differentiation in responding 2° effector CD8 T cells.

In order to determine if extended IL-2 signaling accounts for increased levels of 2° effector CD8 T cell expansion, we neutralized endogenous IL-2 using a blocking antibody, JES6-1A12 [49]. Importantly, the number of accumulated 2° effector P14 CD8 T cells in ‘early’ mice that had received the blocking antibody was significantly reduced (Fig 6G). These data also correlated with a reduced ability of these proliferating cells to incorporate BrdU (Fig 6H). However, this dampened 2° CD8 T cell response observed in the ‘early’ group following IL-2 blockade was not the result of a triggered regulatory CD4 T cell response. While IL-2 bound to the JES6-1A12 monoclonal antibody in a complex selectively stimulates CD25^+^ regulatory CD4 T cells ([49] and S6A and S6B Fig) and increases their proliferation [51], the administration of the blocking antibody alone did not impact regulatory CD4 T cells in both ‘early’ and ‘late’ groups of mice (S6C Fig). Furthermore, administration of the IL-2 blocking antibody resulted in no effect on 2° effector CD8 T cell accumulation or Brdu incorporation within the ‘late’ group (Fig 6I and 6J). These data suggest that since sensitivity to IL-2 signals was already diminished in 2° effector CD8 T cells in the ‘late’ group, neutralization of endogenous IL-2 did not result in any further changes. Thus, decreasing the sensitivity of 2° effector CD8 T cells to IL-2 signaling results in reduced accumulation upon secondary antigen encounter.

Collectively these data suggest that IL-2 signaling regulates levels of 2° expansion and 2° CD8 T cell differentiation, a previously unrecognized mechanism controlling the generation of 2° CD8 T cell responses.

## Discussion

While the formation of 1° M CD8 T cells has been comprehensively studied, much less is known about the factors influencing the generation of 2° M CD8 T cell responses. Longitudinal analyses of developing 1° M and 2° M CD8 T cells suggests that the rate of acquiring a long-term memory phenotype and function varies substantially between these two populations of cells [2]. Prime-boost protocols are often implemented to increase the overall numbers of memory CD8 T cells, leading to the generation of memory CD8 T cells that have encountered Ag more than once. Therefore, understanding if the factors that impact the differentiation of 1° M CD8 T cells also influence 2° M CD8 T cell responses has implications in the design of future consecutive prime-boost vaccine protocols. Here, we show that during an infection recruitment of pathogen-specific 1° M CD8 T cells is not simultaneous, and timing of entry into an immune response relative to the onset of infection impacts the outcome of the ensuing 2° M CD8 T cell response. Specifically, late-entry of 1° M CD8 T cells into the immune response facilitates accelerated acquisition of 2° M characteristics. We also show that the timing of stimulation of 1° M CD8 T cells differentially regulates IL-2 signaling in differentiating 2° effector CD8 T cells, suggesting that this signaling mechanism contributes to the programming of 2° CD8 T cell responses.

D’Souza and Hedrick previously showed that naïve TCR-Tg CD8 T cells are recruited over time after infection with a live replicating pathogen *in vivo* [23]. In another study by Fousteri et al., early and late recruitment of naïve TCR-Tg CD8 T cells was modeled using an adoptive transfer system [24]. Kinetic analyses of Ag-specific CD8 T cells in these studies revealed that the timing of stimulation of naïve CD8 T cells impacted the magnitude of proliferative expansion. Moreover, Bousso et al. demonstrated using adoptive transfer experiments that the timing of entry into an immune response dramatically impacts CD8 T cell proliferative expansion [52]. Thus, Ag-specific CD8 T cells entering the immune response late undergo limited expansion. Additionally, it was observed that the timing of stimulation impacts the rate of 1° M CD8 T cell differentiation, with late-entry into the immune response facilitating faster re-expression of CD62L in Ag-specific CD8 T cells. Interestingly, it has also been shown that the time course of Ag presentation impacts the formation of 1° M CD8 T cells. Van Fessen et al. showed that the fate of differentiating CD8 T cells was heavily influenced by the strength and duration of stimuli [53]. Specifically, naïve CD8 T cells activated in an environment where the strength and duration of stimuli was reduced, preferentially upregulated CD62L [53]. Similarly, it was found by Sarkar et al. that the rate of CD62L re-expression was more rapid in P14 CD8 T cells that were exposed to limited stimulation during LCMV infection [54]. These results suggest that environmental signals as well as the strength of stimuli received by naïve Ag-specific CD8 T cells during an immune response influences 1° M CD8 T cell generation and differentiation. Thus, naïve Ag-specific CD8 T cells entering an immune response early or late may be receiving unique environmental cues at the time of activation, thereby influencing the rate at which these cells acquire 1° M CD8 T cell characteristics.

Our lab has recently shown that similar to 1° M CD8 T cells, 2° M CD8 T cell responses are impacted by the type and duration of infection and inflammatory environment [28,29]. Wirth et al. found that infection with either Vaccinia virus, Att *L*. *monocytognes*, or virulent *L*. *monocytogenes* differentially regulated the kinetics, magnitude, and phenotype of 1° and 2° CD8 T cells in the same host [28]. Additionally in this study it was observed using a peptide-coated DC vaccination strategy that 2° effector CD8 T cells undergo robust proliferation in the presence of systemic inflammation, suggesting that similar to 1° CD8 T cells, 2° CD8 T cell expansion is controlled by the presence of inflammatory cytokines [28]. Given these results, we determined whether the timing of stimulation of 1° M CD8 T cells controls the generation of 2° M CD8 T cell responses. We found that the timing of entry of 1° M CD8 T cells into an immune response impacted the rate at which 2° CD8 T cells transitioned into a long-term 2° M population of cells, however it still remained to be determined what mechanism controlled this unique pattern of differentiation in 2° CD8 T cells.

It has been previously observed that sensitivity to IL-2 signaling at the time of activation impacts 1° CD8 T cell differentiation [49,55,56]. As an example, Williams et al. showed that P14 cells lacking CD25 responded to LCMV infection and developed into populations of long-term memory cells [57]. Interestingly, these CD25-deficient P14 cells readily re-expressed CD62L and CD127, indicating accelerated 1° M CD8 T cells development. However, upon Ag re-encounter these CD25-deficient 1° M P14 cells failed to undergo 2° expansion [57], suggesting that CD25-mediated IL-2 signals during initial activation of CD8 T cells are necessary for 2° responses. In studies by Kalia et al. and Pipkin et al., it has been documented that enhanced IL-2 signaling during activation promotes effector differentiation in 1° CD8 T cell responses [31,32]. Similarly, it was found by Obar et al. that IL-2 signals are important in mediating the formation of effector CD8 T cells with a KLRG1^hi^CD127^lo^ phenotype [58]. We observed that the timing of stimulation of 1° M CD8 T cells modulated the expression of CD25 on 2° effector CD8 T cells, while CD122 expression remained unchanged. This suggested that susceptibility to IL-2 signaling might be an underlying mechanism controlling the programming of 2° M CD8 T cells. Interestingly, we found that 1° M CD8 T cells activated late in the immune response have decreased sensitivity to IL-2 signaling and undergo reduced 2° proliferative expansion. Yet, the transition of these cells to a long-term 2° M phenotype is accelerated. However, enhanced sensitivity to IL-2 signaling following administration of stimulatory IL-2/S4B6 complexes resulted in increased CD25 expression on 2° effector CD8 T cells and favored terminal differentiation (KLRG1^hi^CD127^lo^ phenotype). These data suggest that sensitivity to IL-2 signaling contributes to the development of 2° CD8 T cell responses.

In summary, our data suggests that the process of 1° M to 2° M CD8 T cell differentiation can be manipulated, a notion with great relevance for the generation of 2° M CD8 T cells and the design of consecutive prime-boost vaccine strategies intended to elicit secondary immune responses.

## Materials and Methods

### Ethics statement

All experimental procedures utilizing mice were approved by the University of Iowa Animal Care and Use Committee under the ACURF protocol number 1202050. The experiments performed in this study were done under strict accordance to the Office of Laboratory Animal Welfare guidelines and the PHS Policy on Humane Care and Use of Laboratory Animals.

### Mice and pathogens

C57BL/6 (B6; Thy1.2/1.2) and P14 and OT-I (Thy1.1; specific for lymphocytic choriomeningitis virus (LCMV)-derived GP33 and chicken ovalbumin Ova257 epitopes, respectively) T-cell-receptor transgenic (TCR-Tg) mice were bred at the University of Iowa and housed under pathogen-free conditions. All mice were used at 6–10 weeks of age. All animal procedures followed approved Institutional Animal Care and Use Committee (ACURF) protocols. The Armstrong strain of LCMV (LCMV, 2x10^5^ PFU/mouse, i.p.) and attenuated *actA*-deficient *L*. *monocytogenes* strain expressing Ova257 (Att LM-Ova, 5x10^6^ CFU/mouse, i.v.) or GP33 (Att LM-GP33, 1x10^7^ CFU/mouse, i.v.) were grown, injected, and quantified as described [59,60]. Infected mice were housed at the University of Iowa under the appropriate biosafety level.

### Adoptive transfer and isolation of lymphocytes from tissues

For 1° CD8 T cell responses, naïve Thy1.1 P14 or OT-I cells were obtained from peripheral blood of young naïve TCR-Tg P14 or OT-I mice. Contaminating memory phenotype (CD44^hi^CD11a^hi^) P14 and OT-I cells were always <5%. Naïve P14 or OT-I cells were transferred (5x10^3^ P14 or 1x10^3^ OT-I cells/mouse, i.v.) [33] into recipient Thy1.2/1.2 mice on the day of (‘early’ group) or 3 days after (‘late’ group) LCMV or Att LM-Ova infection. To generate 1° M P14 or OT-I cells for adoptive transfer experiments, 1x10^3^ naïve Thy1.1 P14 or OT-I cells were transferred into Thy1.2 recipients and mice were then immunized with either LCMV (2x10^5^ PFU/mouse, i.p.) or Att LM-Ova (5x10^6^ CFU/mouse, i.v.), respectively. At days 40–75 after infection, 1° M P14 or OT-I cells were isolated from spleens of mice by positive selection for Thy1.1 and transferred (2-4x10^4^ cells/mouse, i.v.) into recipient Thy1.2/1.2 mice on the day of (‘early’ group) or 3 days after (‘late’ group) LCMV or Att LM-Ova infection.

Before removal of secondary lymphoid organs and tertiary tissues, samples of blood were obtained by retro-orbital puncture. Anesthetized mice were then perfused through the left ventricle with cold PBS and tissues were collected. Single-cell suspension from spleen, lung, and lymph nodes (LN) were washed before Ab staining. For experiments determining the expression of CD25 and CD69 on CFSE-labeled P14 CD8 T cells, spleens were cut into small pieces and treated with collagenase D (150U/mL) for 30 min at 37°C before further processing [44].

### Antibodies and peptides

Flow cytometry data was acquired on a FACS Canto flow cytometer (Beckton-Dickinson Biosciences) and analyzed with FlowJo software (Tree Star). The following is a list of used mAbs with the indicated specificity and appropriate combinations in flourochromes from eBioscience: CD8 (clone 53–6.7), CD4 (GK1.5) Thy1.1 (HIS51), CD27 (LG.759), CD62L (MEL-14), KLRG1 (2F1), CD25 (PC61.5), CD69 (H1.2F3), CD122 (5H4), Bcl2 (BCL/104C4), Eomes (Dan11mag), Tcf1 (C63D9), Tbet (eBio4B10) BrdU (Bu20a), Foxp3 (FJK-16s) and appropriate isotype controls. Western mAb for Cyclin A (CY-A1; mouse, Sigma), Cyclin B1 (Rabbit, Cell Signaling Technology), FoxM1 (Rabbit, Cell Signaling Technology), and β-actin (Mouse, Santa Cruz Biotechnology) were previously described [48]. Intracellular staining for IFNγ (XMG1.2, Biolegend), TNFα (MP6-XT22, Biolegend), and IL-2 (JES6-5H4, eBioscience) was performed after surface fixation and permeabilization of the cell membrane using cytofix/cytoperm solution. Anti-STAT5 (pY694) was purchased from BD Biosciences. Synthetic GP33-41 peptide was used as previously described [17,61].

### Quantification of CD8 T cell responses

P14 and OT-I cell responses in the peripheral blood and tissues were monitored by FACS analysis for Thy1.1-positive CD8 T cells. Cells were incubated with mAb at 4°C for 30 min, washed with FACS buffer (PBS containing 1% FCS and 0.1% NaN3), and then fixed with cytofix/cytoperm solution. The percentage of CD8 T cells producing cytokines after stimulation with GP33 peptide was determined using intracellular cytokine staining for IFNγ and TNFα or IL-2 after 5 h incubation in brefeldin A with or without GP33 peptide.

### CFSE labeling and BrdU incorporation

For adoptive transfer experiments of CFSE labeled 1° M CD8 T cells, 10^6^ splenocytes/mL from LCMV immune mice, containing 1° M Thy1.1 P14 cells, were incubated for 15 minutes at 37°C in the presence of 5mM CFSE. CFSE-labeled cells were washed twice with PBS containing 10% fetal calf serum and 1x10^6^ 1° M Thy1.1 P14 cells were injected into Thy1.2/1.2 recipient mice. To measure division of 2° effector Thy1.1 CD8 T cells BrdU was injected (2 mg/mouse, i.p.) for ~15 h before spleen harvest. Detection of BrdU incorporation was performed according to manufacturer’s protocol (BrdU Flow kits; BD) [48].

### Quantitative RT-PCR

Spleen cells from ‘early’ and ‘late’ groups were harvested at day 7 post-transfer. 2° effector Thy1.1 P14 CD8 T cells were sorted directly into Trizol LS reagent (Invitrogen). Following chloroform extraction, the aqueous phase was mixed with 2 volumes of ethanol and loaded onto a purification column from the RNeasy Mini Kit (Qiagen) for further purification. Total RNA was reverse-transcribed using QuantiTech Reverse Transcription Kit (Qiagen). The resulting cDNA was analyzed for the expression of different genes by quantitative PCR using SYBR Advantage qPCR pre-mix (Clontech) on an ABI 7300 Real Time PCR System (Applied Biosystems) as previously described [62]. The relative gene expression levels in each sample were normalized to the housekeeping gene, hypoxanthine phosphoribosyltransferase (*Hprt*). The primer sequences include the following:

*Bcl2*: 5’-GCAGATTGCCCTGGATGT-AT and 5’- AGAAAAGTCAGCCAGCCAGA;

*Eomes*: 5’-TCCTAACACTGGCTCCCACT and 5’-GTCACTTCCACGATGTGCAG;

*Tcf7*: 5’-CAATCTGCTCATGCCCTACC and 5’-CTTGCTTCTGGCTGATGTCC;

*Prdm1*: 5’-CCAAGGAACCTGCTTTTCAA and 5’-GGCATTCTTGGGAACTGTGT;

*Id3*: 5’–ATCTCCCGATCCAGACAGC and 5’–GAGAGAGGGTCCCAGAGTCC;

*Tbx21*: 5’-CAATGTGACCCAGATGATCG and 5’-GCGTTCTGGTAGGCAGTCAC;

*Hprt*: 5’-GCGTCGTGATTAGCGATGATG and 5’-CTCGAGCAAGTCTTTCAGTCC.

### *In vitro* IL-2 stimulation

2° effector Thy1.1 P14 cells (2x10^6^) from ‘early’ and ‘late’ groups of mice were incubated *in vitro* in the presence or absence of murine rIL-2 (Peprotech). STAT5-pY694 activation was measured directly following murine rIL-2 stimulation at indicated concentrations using manufacturer’s protocol (BD Phosflow; Cell Signaling) [48].

### Immunoblot analysis

2° effector Thy1.1 P14, from ‘early’ and ‘late’ group of mice as well as 1° M Thy1.1 P14 cells were stained with PE-anti-Thy1.1 (Clone OX-7, BD PharMingen), and purified with PE-antibody magnetic beads according to standard AutoMacs protocols. Cells were then immediately lysed in in NP-40 lysis buffer (20mM Hepes, pH 7.9, 100mM NaCl, 5mM EDTA, 0.5 mM CaCl, 1%NP-40, 1mM PMSF, 10μM MG-132) for 15 minutes on ice as previously described [48,63]. Whole-cell lysates were clarified by centrifugation at ~20,000 *g* for 5 minutes at 4°C. Extracts were then resolved by SDS-PAGE (BIO RAD). Primary antibodies were detected with goat anti-mouse IgG or goat anti-rabbit IgG coupled to horseradish peroxidase (Santa Cruz Biotechnology) and SuperSignal West Pico Chemiluminescence (Thermo Scientific).

### *In vivo* cytokine stimulation/neutralization

IL-2/mAb complexes were generated by incubating murine rIL-2 (Peprotech) with either S4B6 or JES6-1A12 anti-IL-2 monoclonal antibodies at a 2:1 molar ratio (1.5μg/mL IL-2: 50μg/mL S4B6 or JES6-1A12) for 15 minutes at room temperature [48]. IL-2 neutralization was achieved in the ‘early’ group of mice by injecting 500μg JES6-1A12 alone daily from day 2–5 post-transfer. For control, equal amounts of rat IgG (Sigma-Aldrich) were used in an additional group of mice [48].

### Statistical analyses

Data were analyzed with Prism4 GraphPad software to determine statistical significance as indicated in figure legends. Statistical significance was assessed using the two-tailed, unpaired student’s T test, with a confidence interval >95% (*p ≤ 0.05, **p ≤ 0.005, ***p ≤ 0.001. and n.s. as no significance). Data generated as scatter dot plots are presented as mean, and data generated as bar graphs are presented as mean + SEM.

## Supporting Information

S1 Fig Timing of stimulation of naïve CD8 T cells impacts 1° M CD8 T cell differentiation. **A)** Experimental design. Naïve B6 Thy1.2/1.2 mice received a transfer of naïve Thy1.1 P14 CD8 T cells (5x10^3^ cells/mouse, i.v.) on the day of (‘early’ group) or 3 days after (‘late’ group) infection with LCMV (2x10^5^ PFU/mouse, i.p.). **B)** The percentage of 1° effector P14 CD8 T cells in the PBL at day 8 after transfer. Dots represent individual mice and the line represents the mean. **C)** The percentage of 1° M P14 CD8 T cells in the PBL 1 month after transfer. **D)** Blood samples were pooled, and representative histograms show the expression of the molecules CD27, CD62L, and KLRG1 on 1° M P14 CD8 T cells 1 month after transfer. Shaded graphs represent isotype control staining and open graphs represent specific Ab staining on gated 1° M Thy1.1 P14 CD8 T cells. **E)** The percentage of 1° M P14 CD8 T cells in the PBL 6 months after transfer. **F)** Blood samples were pooled, and representative histograms show the expression of CD27, CD62L, and KLRG1 on 1° M P14 CD8 T cells in the PBL 6 months after transfer. **G)** The percentage of Thy1.1 P14 CD8 T cells in the PBL of individual mice from ‘early’ and ‘late’ groups was determined at indicated days after transfer and then normalized to the peak of response (day 8). **H)** The percentage of *L*. *monocytogenes*-specific Thy1.1 OT-I CD8 T cells in the PBL of individual mice from ‘early’ and ‘late’ groups was determined at indicated days after transfer and then normalized to the peak of the response (day 7). Dots represent individual mice.
(TIF)

S2 Fig Late stimulated 1° M CD8 T cells progress to 2° M at an accelerated rate. Kinetic analysis of the expression of CD27, CD62L, and KLRG1 molecules on 2° P14 CD8 T cells from pooled blood samples from ‘early’ and ‘late’ groups of mice on various days after transfer. Data are presented as the percentage of positive cells for the indicated marker.
(TIF)

S3 Fig Timing of simulation of 1° M OT-I cells impacts 2° M CD8 T cell differentiation. **A)** Experimental design. Naïve B6 Thy1.2/1.2 mice received a transfer of 1° M Thy1.1 OT-I CD8 T cells (4x10^4^ cells/mouse, i.v.) on the day of (‘early’ group) or 3 days after (‘late’ group) infection with Att LM-Ova (5x10^6^ CFU/mouse i.v.). **B)** The percentage of 2° effector OT-I CD8 T cells in the PBL at day 6 after transfer. Dots represent individual mice and the line represents the mean. **C)** Kinetic analysis of the 2° OT-I CD8 T cell response over time. Data are presented as the percentage (mean + SD for 7–10 mice per group, per time point) of OT-I CD8 T cells in the PBL of mice at indicated days after transfer. **D)** Blood samples were pooled, and representative histograms show the expression of the molecules CD27, CD62L, and KLRG1 on 2° M OT-I CD8 T cells in the PBL at day 35 after transfer. Shaded graphs represent isotype control staining and open graphs represent specific Ab staining on gated 2° M Thy1.1 P14 CD8 T cells.
(TIF)

S4 Fig Timing of stimulation does not impact CD122 expression on responding 2° CD8 T cells. Representative histograms showing the expression of the CD122 molecule on 2° effector P14 CD8 T cells isolated from the spleens of ‘early’ and ‘late’ groups of mice on the indicated days after transfer. Shaded graphs represent isotype control staining and open graphs represent specific Ab staining on gated 2° effector P14 CD8 T cells. Black numbers indicate the percentage of P14 CD8 T cells positive for CD122.
(TIF)

S5 Fig Antigenic stimulation and increased sensitivity to IL-2 signals facilitate 2° CD8 T cell expansion. **A)** Naïve B6 Thy1.2/1.2 mice received a transfer of 1° M P14 CD8 T cells (2x10^4^/mouse, i.v.) on the day of (‘early’ group) or 3 days after (‘late’ group) infection with LCMV, or were left uninfected (w/o infection group). The percentage of P14 CD8 T cells was then determined in the spleens of individual mice from ‘early,’ ‘late,’ and w/o infection groups on day 7 post transfer. Data are presented as mean+ SEM of 4–5 mice per group.
(TIF)

S6 Fig Neutralizing endogenous IL-2 signals during 2° expansion phase does not impact the percentage of Tregs. **A-B)** ‘Early’ groups of mice were treated with control IgG or IL-2/JES6 stimulating complex (1.5μg/mL IL-2: 50µg/mL JES6-1A12) from days 2–5 post-transfer. **A)** Representative dot plots showing the percentage of Foxp3+ CD4 T cells in the spleen of individual mice in the ‘early’ group. **B)** The percentage of FoxP3^+^ CD4 T cells (Treg) from the spleens of individual mice in the ‘early’ group at day 7 post transfer is shown. **C)** ‘Early’ and ‘late’ groups of mice were treated with control IgG or IL-2 blockade (JES6-1A12, 500 μg/mouse) from days 2–5 post transfer. The percentage of FoxP3+ CD4 T cells from the spleens of individual mice in ‘early’ and ‘late’ groups at day 7 post transfer is shown. Data are presented as mean+SEM of 4 mice per group.
(TIF)
