# Supplementary figures and images for: The Timing of Stimulation and IL-2 Signaling Regulate Secondary CD8 T Cell Responses
Source: PLoS Pathog. 2015 Oct 2;11(10):e1005199. doi: 10.1371/journal.ppat.1005199 (PMC4592272; doi:10.1371/journal.ppat.1005199)

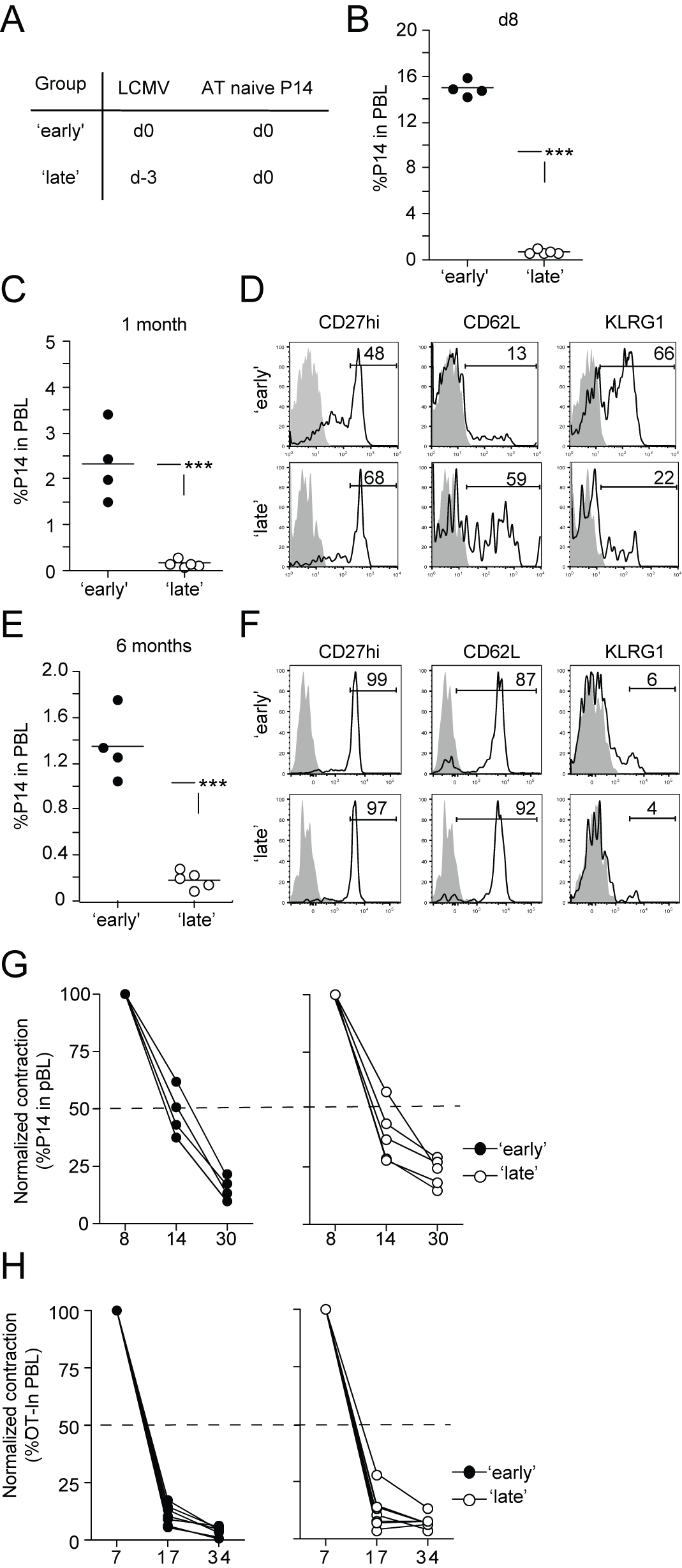

Supplement: S1 Fig — A) Experimental design. Naïve B6 Thy1.2/1.2 mice received a transfer of naïve Thy1.1 P14 CD8 T cells (5x10^3^ cells/mouse, i.v.) on the day of (‘early’ group) or 3 days after (‘late’ group) infection with LCMV (2x10^5^ PFU/mouse, i.p.). B) The percentage of 1° effector P14 CD8 T cells in the PBL at day 8 after transfer. Dots represent individual mice and the line represents the mean. C) The percentage of 1° M P14 CD8 T cells in the PBL 1 month after transfer. D) Blood samples were pooled, and representative histograms show the expression of the molecules CD27, CD62L, and KLRG1 on 1° M P14 CD8 T cells 1 month after transfer. Shaded graphs represent isotype control staining and open graphs represent specific Ab staining on gated 1° M Thy1.1 P14 CD8 T cells. E) The percentage of 1° M P14 CD8 T cells in the PBL 6 months after transfer. F) Blood samples were pooled, and representative histograms show the expression of CD27, CD62L, and KLRG1 on 1° M P14 CD8 T cells in the PBL 6 months after transfer. G) The percentage of Thy1.1 P14 CD8 T cells in the PBL of individual mice from ‘early’ and ‘late’ groups was determined at indicated days after transfer and then normalized to the peak of response (day 8). H) The percentage of L. monocytogenes-specific Thy1.1 OT-I CD8 T cells in the PBL of individual mice from ‘early’ and ‘late’ groups was determined at indicated days after transfer and then normalized to the peak of the response (day 7). Dots represent individual mice. (TIF) [file ppat.1005199.s001.tif]

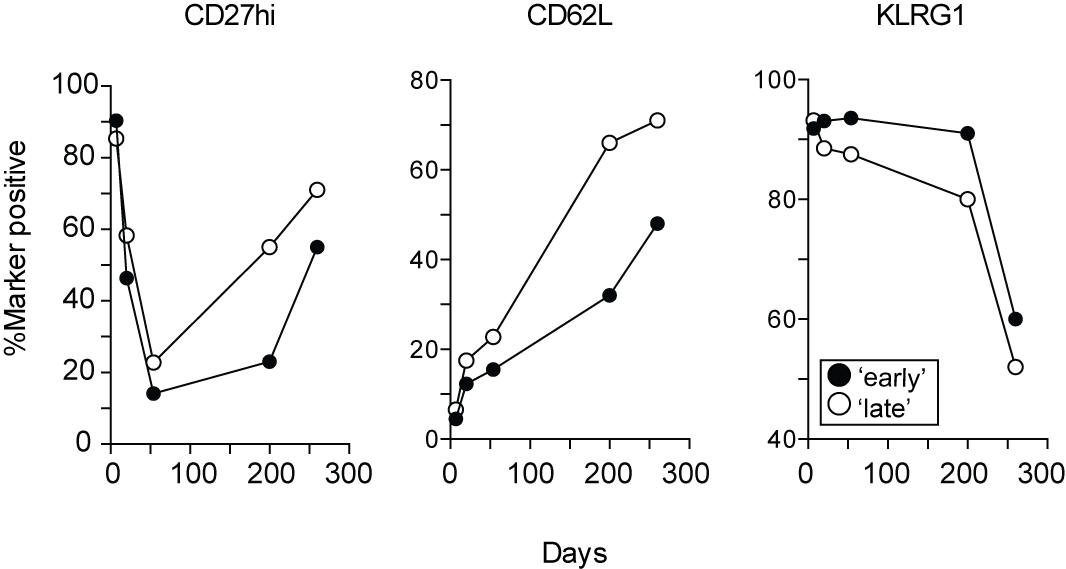

Supplement: S2 Fig — Kinetic analysis of the expression of CD27, CD62L, and KLRG1 molecules on 2° P14 CD8 T cells from pooled blood samples from ‘early’ and ‘late’ groups of mice on various days after transfer. Data are presented as the percentage of positive cells for the indicated marker. (TIF) [file ppat.1005199.s002.tif]

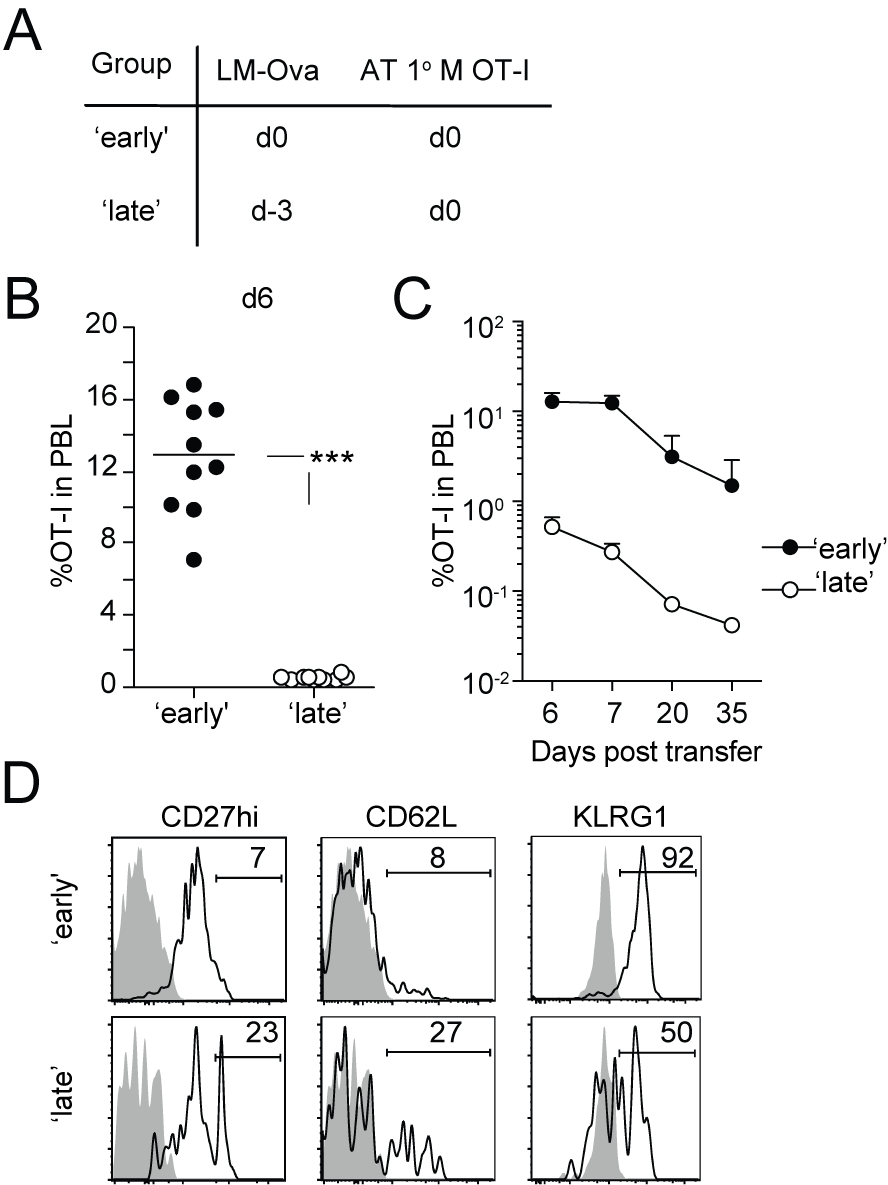

Supplement: S3 Fig — A) Experimental design. Naïve B6 Thy1.2/1.2 mice received a transfer of 1° M Thy1.1 OT-I CD8 T cells (4x10^4^ cells/mouse, i.v.) on the day of (‘early’ group) or 3 days after (‘late’ group) infection with Att LM-Ova (5x10^6^ CFU/mouse i.v.). B) The percentage of 2° effector OT-I CD8 T cells in the PBL at day 6 after transfer. Dots represent individual mice and the line represents the mean. C) Kinetic analysis of the 2° OT-I CD8 T cell response over time. Data are presented as the percentage (mean + SD for 7–10 mice per group, per time point) of OT-I CD8 T cells in the PBL of mice at indicated days after transfer. D) Blood samples were pooled, and representative histograms show the expression of the molecules CD27, CD62L, and KLRG1 on 2° M OT-I CD8 T cells in the PBL at day 35 after transfer. Shaded graphs represent isotype control staining and open graphs represent specific Ab staining on gated 2° M Thy1.1 P14 CD8 T cells. (TIF) [file ppat.1005199.s003.tif]

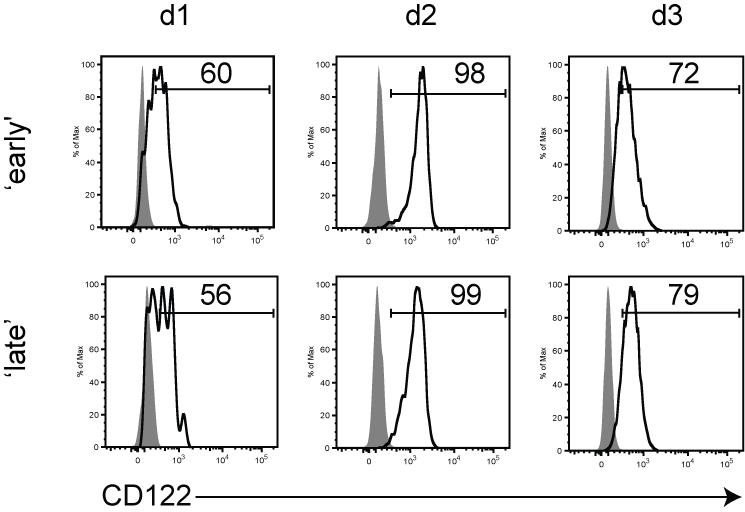

Supplement: S4 Fig — Representative histograms showing the expression of the CD122 molecule on 2° effector P14 CD8 T cells isolated from the spleens of ‘early’ and ‘late’ groups of mice on the indicated days after transfer. Shaded graphs represent isotype control staining and open graphs represent specific Ab staining on gated 2° effector P14 CD8 T cells. Black numbers indicate the percentage of P14 CD8 T cells positive for CD122. (TIF) [file ppat.1005199.s004.tif]

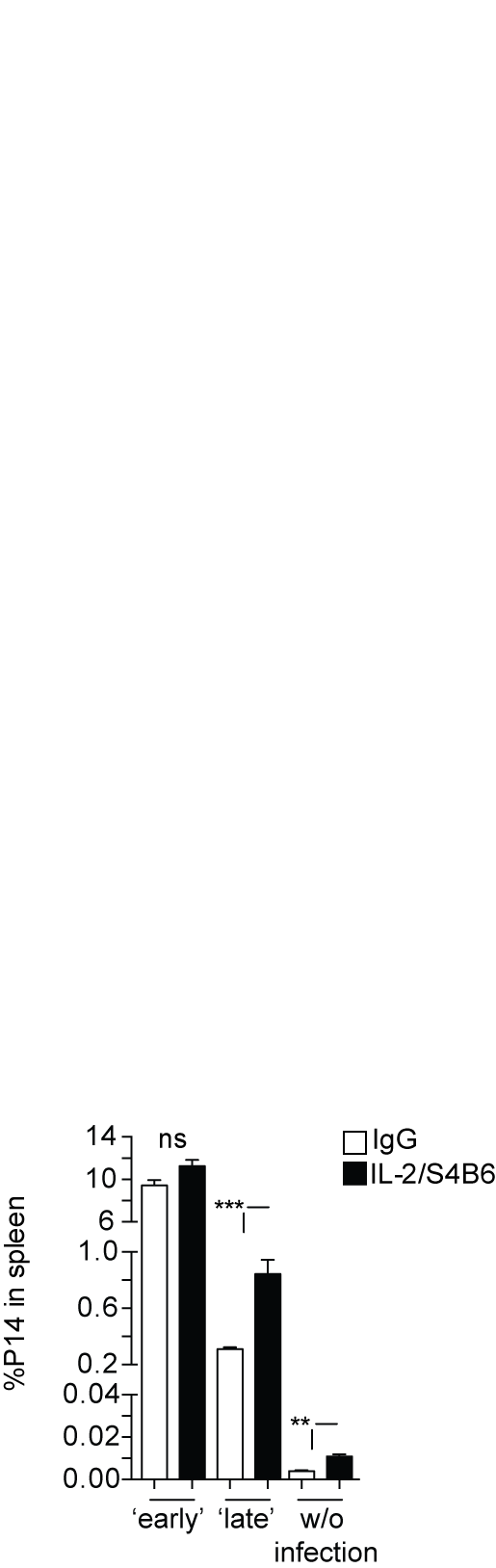

Supplement: S5 Fig — A) Naïve B6 Thy1.2/1.2 mice received a transfer of 1° M P14 CD8 T cells (2x10^4^/mouse, i.v.) on the day of (‘early’ group) or 3 days after (‘late’ group) infection with LCMV, or were left uninfected (w/o infection group). The percentage of P14 CD8 T cells was then determined in the spleens of individual mice from ‘early,’ ‘late,’ and w/o infection groups on day 7 post transfer. Data are presented as mean+ SEM of 4–5 mice per group. (TIF) [file ppat.1005199.s005.tif]

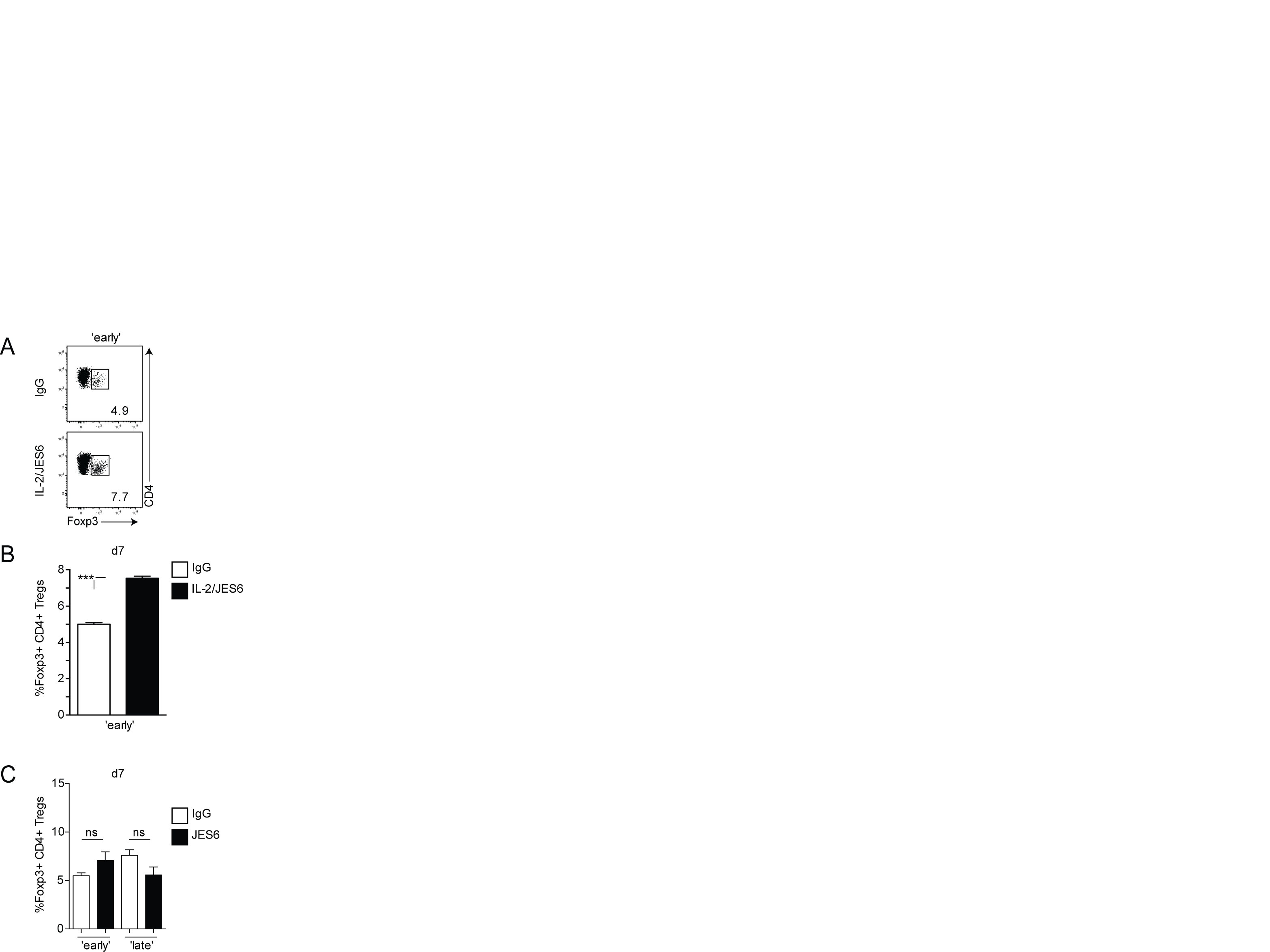

Supplement: S6 Fig — A-B) ‘Early’ groups of mice were treated with control IgG or IL-2/JES6 stimulating complex (1.5μg/mL IL-2: 50µg/mL JES6-1A12) from days 2–5 post-transfer. A) Representative dot plots showing the percentage of Foxp3+ CD4 T cells in the spleen of individual mice in the ‘early’ group. B) The percentage of FoxP3+ CD4 T cells (Treg) from the spleens of individual mice in the ‘early’ group at day 7 post transfer is shown. C) ‘Early’ and ‘late’ groups of mice were treated with control IgG or IL-2 blockade (JES6-1A12, 500 μg/mouse) from days 2–5 post transfer. The percentage of FoxP3+ CD4 T cells from the spleens of individual mice in ‘early’ and ‘late’ groups at day 7 post transfer is shown. Data are presented as mean+SEM of 4 mice per group. (TIF) [file ppat.1005199.s006.tif]
